# A Human Curiosity

**Published:** 1866-07

**Authors:** 


A Human Curiosity.—The Berlin newspapers have the following curious paragraph: "A Hungarian girl, born at Oedenburg, without hands, now twenty years of age, has been giving some curious representations in the Prussian capital. She performs with her mouth the functions of her hands. She sews, embroiders, executes the most delicate work with pearls, even threads her needles and makes knots, all with the tongue, apparently without difficulty, and certainly without the assistance of any one. Part of the works thus executed are destined for public exhibition." Most people will hesitate to believe such marvels until they witness them.

